# Predictive Models of Odor Contribution and Thresholds for Volatiles in Identification of Novel Crop Aroma Compounds

**DOI:** 10.3390/metabo15110747

**Published:** 2025-11-18

**Authors:** Qiao Li, Shaofang Li, Jie Luo, Honglun Yuan

**Affiliations:** 1 State Key Laboratory of Tropical Crop Breeding, School of Breeding and Multiplication (Sanya Institute of Breeding and Multiplication), Hainan University, Sanya 572025, China; 19529745446@163.com (Q.L.); 17854561863@163.com (S.L.); 2 School of Tropical Agriculture and Forestry, Hainan University, Haikou 570288, China; 3 Yazhouwan National Laboratory, Sanya 572025, China

**Keywords:** volatiles, machine-learning, odor contribution, odor threshold, passion fruits

## Abstract

**Background/Objectives**: Aroma is a key determinant of crop quality and consumer acceptance, and aroma contribution and odor threshold are critical attributes for the identification of aroma compounds. Because the experimental determination of aroma contribution and odor thresholds is time-consuming and complex, most volatiles lack contribution and/or threshold data. **Methods**: We compiled odor thresholds for 716 volatile compounds and 31,459 aroma contribution records, and trained machine-learning models that took molecular fingerprints and physicochemical descriptors (e.g., molecular weight, logP, TPSA) as inputs to predict aroma contribution and odor threshold. We evaluated multiple fingerprint–model combinations, optimized hyperparameters via 5-fold cross-validation on the training set, and assessed the best models on a held-out validation set. **Results**: The ECFP6–GBDT combination performed best for predicting aroma contribution (macro-F1 = 0.732; weighted-F1 = 0.912). The ECFP4–GBDT model performed best for predicting odor thresholds (R^2^ = 0.94; RMSE = 0.44). Applying the models to volatiles in passion fruit juice identified 2-phenylethyl acetate as a potential new contributor to passion fruit aroma, whereas menthyl acetate likely exerted a negative influence; both findings were confirmed by serial dilution and sensory evaluation. The developed models provided both a GUI and a CLI, were easy to use, and supported straightforward upgrades by retraining with user-provided data. **Conclusions**: This work provided a methodological foundation for identifying crop aroma compounds and supported the genetic improvement of aroma traits.

## 1. Introduction

Aroma is a salient and memorable dimension of crop quality that strongly shapes consumer liking and repeat purchase [1]. Cultivars with distinctive and pleasant bouquets typically command price premiums and facilitate branding and geographical indication strategies. A study on strawberry consumer preferences found that flavor intensity was strongly associated with consumer liking, whereas Tieman and colleagues reported that modern tomatoes contained reduced levels of multiple flavor compounds, leading to significantly lower consumer ratings [2,3]. In real markets, varieties with signature aroma profiles can achieve price uplifts. For example, aromatic rice fetches a premium price [4]. Therefore, systematically resolving the chemical basis of crop aroma—i.e., which volatiles drive perceived quality and by what mechanisms—is both scientifically meaningful and practically valuable.

Solid-phase microextraction (SPME) gas chromatography-mass spectrometry (GC-MS)-based untargeted volatilomics is the most commonly used method to profile volatiles of plant [5,6,7]. Our group previously developed the widely targeted volatilomics (WTV) series that extended detection and annotation coverage [8,9]. The WTV series of methods were applied to volatile profiling across diverse crops, enabling researchers to more comprehensively characterize crop volatile compositions [10,11,12,13,14,15,16,17]. However, translating a detected-compound list into causal odorants requires two critical parameters for each volatile—odor threshold and aroma contribution. In their absence, one cannot reliably distinguish volatiles that are merely present from those that, even at low abundance, dominate perception.

Here, odor threshold refers to the minimum concentration at which a volatile is unambiguously perceived in a specified matrix (air/water/oil) [18], often used to compute the odor activity value (OAV = concentration/threshold). Aroma contribution denotes the direction and magnitude of a volatile’s effect on the overall aroma—positive (e.g., fruity, floral, creamy) or negative (e.g., musty, moldy, sulfurous). These attributes are determined by sensory evaluation with trained panels or by instrumental analysis [19,20]. However, these approaches are labor-intensive and costly, sensitive to panelist training and day-to-day condition, and confounded by matrix and environmental factors; consequently, reproducibility and cross-laboratory transferability can be limited [21]. This bottleneck explains why many detected volatiles remain unannotated in sensory terms.

Studies in related fields have demonstrated that structure-to-odor prediction is feasible at scale [21,22,23,24,25,26], and this has greatly improved the accuracy and efficiency of flavor-related research across domains [27,28]. In drinking-water off-flavor compound identification, a machine-learning model linking molecular structure (or MS^2^ spectra) to odor perception/threshold was developed, and it was able to streamline the odor-attribute prediction and represented a crucial advancement toward credible tracking and efficient control of off-odors [21]. In perfume research, investigators developed a deep-learning framework with transfer learning. A graph convolutional network (GCN) first predicted semantic odor descriptors; its learned representations were then transferred as features to a feed-forward neural network (FNN) that estimated odor thresholds from molecular structure [25]. The model served as a powerful tool to screen compounds suitable for the perfumery. Collectively, these advances supported the practicality of predicting odor thresholds and contributions from molecular structure.

In this study, we developed a machine-learning model that leveraged molecular fingerprints and physicochemical descriptors to predict the aroma contribution and odor thresholds of crop volatiles. Bench validation indicated reliable performance, and application to passion fruit volatilomes nominated previously unrecognized candidates that might influence its aroma. The models provided both a graphical user interface (GUI) and a command-line interface (CLI), and served as a methodological foundation for aroma compound identification and aroma-trait improvement in crop breeding.

## 2. Materials and Methods

### 2.1. Plant Materials

Mature fruits of passion fruit (*Passiflora edulis* Sims) cultivar Tainong were purchased from local market. Three biological replicates were utilized, each consisting of six fruits.

### 2.2. Chemical Reagents

The authentic standards were purchased from Shanghai Aladdin Biochemical Technology Co. (Shanghai, China; https://www.aladdin-e.com/, accessed on 13 November 2025) and Sigma-Aldrich (St. Louis, MO, USA; https://www.sigmaaldrich.cn/CN/zh, accessed on 13 November 2025). The C8-C20 alkane standard mix solution (~40 mg/L each, in hexane) was purchased from Sigma-Aldrich.

### 2.3. Sample Preparation

We added 1.2 g NaCl into each 4 mL passion fruit juice sample and equilibrated for 30 min. Then, the sample was subjected to SPME (Agilent, Santa Clara, CA, USA). The desorbed compounds were then injected into the GC-MS 7890–7000D (Agilent, Santa Clara, CA, USA) for analysis [9].

### 2.4. GC–MS Conditions

GC-MS analysis on volatiles was performed according to our previously described method [9]. Passion fruit juice was preheated for 10 min at 40 °C and extracted for 20 min at 40 °C. The initial temperature of the oven was set at 40 °C and maintained for 5 min. It was then increased to 270 °C at a rate of 5 °C/min, followed by a further increase to 300 °C at a rate of 25 °C/min. The temperature was maintained at 300 °C for 5 min. To determine the retention index, an alkyl standard of C8∼C20 was used under the same temperature program. For widely targeted profiling, selected ion monitoring (SIM) mode was used, and the MS settings were configured based on the results of the method generator.

### 2.5. Metabolome Data Analysis

Qualitative analysis was carried out using the *data analyzer* module of WTV 2.0. The parameters were set as follows: smooth factor: 5, peak filter factor: 10, and bin number: 0.5; using RI mode: library search window: 100, maximum RI: 3000, match weight: 0.70, reverse match weight: 0.30, minimum ion number in component for identification: 1, and similarity score threshold: 0.40; and calculate RI penalty: RI window: ±20.00, RI window scale: 2.00, level factor: 0.05, maximum penalty: 0.20, no RI penalty: 0.15, inaccurate RI threshold: 800, and inaccurate RI level factor: 0.01.

### 2.6. Aroma Contribution and Odor Threshold Data Collection

We aggregated odor-descriptor annotations for 31,459 volatile compounds from multiple databases [29,30,31,32]. For each compound, synonymous descriptors were first consolidated. We then grouped descriptors into higher-level semantic categories (e.g., *fruit*, *fruity*, *apple*, *pear* → Fruity). When a compound’s descriptors mapped to multiple categories, the final category was assigned by a fixed priority scheme (e.g., Fruity had higher priority than Green/Herbal). To increase sample sizes and reflect similar sensory impacts, we merged sparse or closely related categories into broader groups (e.g., Dairy/Buttery into Sweet/Vanilla/Caramel; Rancid/Sweaty/Animalic and Chemical/Solvent/Plastic into Off-flavor). We then assigned contribution labels at the category level (e.g., Fruity and Citrus as positive; Off-flavor and Odorless as negative). Detailed rules for category consolidation and contribution labeling are provided in Supplementary Table S1.

Odor thresholds were compiled for 716 volatiles. When multiple threshold measurements existed for a compound, we used the median value. All thresholds were harmonized to mg/L and transformed to negative base-10 logarithms (−log10[mg/L]) for analysis.

### 2.7. Model Development and Validation

When inputs were provided as compound names, we converted them to canonical SMILES and deduplicated records by SMILES. We performed a Murcko scaffold-aware greedy split to create a training set and a held-out validation set (default validation fraction 0.2). To mitigate class imbalance, the majority class in the training set was downsampled to 5,000 instances (as appropriate for this dataset). We computed three molecule-level fingerprints from SMILES—ECFP4 (1024 bits), ECFP6 (1024 bits), and MACCS (167 bits)—for use with RF, GBDT, and MLP models, and constructed molecular graphs for a GCN model. We also calculated physicochemical descriptors (molecular weight, logP, topological polar surface area, and molar refractivity) and concatenated them with the fingerprint or graph-based features as model inputs. We carried out a randomized hyperparameter search with 5-fold cross-validation on the training set, using macro-F1 (mean across folds) to select the best configuration for each model family. The searched spaces are as follows:

RF:clf__n_estimators ∈ {200, 300, 400, 600}clf__max_depth ∈ {10, 20, 30}clf__min_samples_split ∈ {2, 5, 10}clf__min_samples_leaf ∈ {1, 2, 4}clf__max_features ∈ {“sqrt”, “log2”}GBDT (gradient-boosted trees):clf__n_estimators ∈ {300, 600, 900}clf__max_depth ∈ {4, 6, 8}clf__learning_rate ∈ {0.03, 0.05, 0.1}clf__subsample ∈ {0.7, 0.9, 1.0}clf__colsample_bytree ∈ {0.6, 0.8, 1.0}clf__reg_lambda ∈ {0.0, 1.0, 3.0}MLP:clf__hidden_layer_sizes ∈ {(512,128), (256,128), (256,64)}clf__alpha (L2) from 5 log-spaced values between 1 × 10^−5^ and 1 × 10^−3^clf__learning_rate_init from 5 log-spaced values between 1 × 10^−4^ and 1 × 10^−3^clf__batch_size ∈ {128, 256, 512}GCN:hidden dimension ∈ {64, 128, 256}; dropout ∈ {0.1, 0.3, 0.5};learning rate ∈ {1 × 10^−3^, 3 × 10^−3^, 5 × 10^−4^}; weight decay ∈ {0.0, 1 × 10^−4^, 5 × 10^−4^};batch size ∈ {64, 128, 256}.

After selecting the best hyperparameters per model, we retrained on the full training set and evaluated each fingerprint–model combination on the held-out validation set. We ranked combinations primarily by validation macro-F1 and broke ties by Accuracy, then Macro-Precision, then Macro-Recall. The globally best configuration was retained for downstream use.

For threshold modeling, inputs consisted of SMILES and measured thresholds (mg/L). We removed entries with missing SMILES or thresholds, excluded thresholds ≤ 0, and transformed thresholds to −log10[mg/L]. SMILES were canonicalized and deduplicated; for duplicates, we averaged multiple threshold values per compound. Data splitting, feature construction, and model families matched the category/contribution workflow. Hyperparameter selection used validation R^2^ as the primary criterion and RMSE as a tie-breaker; other procedures were identical. To examine performance across different threshold levels, we partitioned the validation set thresholds into tertiles—Low (≤q1), Mid (q1–q2), and High (>q2)—where q1 and q2 were the 1/3 and 2/3 quantiles of the validation thresholds. We reported RMSE within each tertile.

### 2.8. Serial Ddilution and Sensory Evaluation

Sensory detection thresholds were measured in a distilled-water matrix using an ascending concentration series method of limits based on ASTM E679 (forced-choice ascending concentration series) with minor modifications. Specifically, we used a single-bottle yes/no detection task rather than 3-alternative forced-choice (3-AFC) sets, and defined group thresholds at 50% detection. All tests were conducted in distilled water (matrix = water; no juice or sugar/acid additions).

Stock solutions of 2-phenylethyl acetate and menthyl acetate were prepared in water and then serially diluted 10-fold to produce graded concentrations (e.g., 0.1, 1, 10, 100, 1000) along with a blank. For each sample, 1–2 mL was aliquoted into 2 mL amber headspace/GC vials sealed with PTFE/silicone septa and labeled with three-digit random codes. Within each session, samples were presented in randomized blocks or Latin-square order; assessors were instructed to begin with the blank and the lowest concentration and proceed upward to reduce adaptation and contrast effects. Evaluations were performed in controlled sensory booths (24 ± 1 °C; 45–55% relative humidity; neutral white lighting) with minimal background odor. Samples were equilibrated at room temperature for 10–15 min prior to assessment.

The panel comprised 20 assessors experienced in aroma description (n = 20). At the start of each session, assessors briefly calibrated and anchored a 0–5 intensity scale (0 = none; 5 = very strong) using low/mid/high items from the dilution series (e.g., tubes 3, 2, and 1). Assessors refrained from flavored drinks and fragrances for ≥1 h before testing. Orthonasal sniffing was used (2–3 s per vial), with ≥30 s rest between samples and water provided for neutralization. A blank was reinserted after every 3–4 samples to monitor carryover. Each coded sample was rated within the session by all assessors and, when necessary, across multiple sessions.

For each coded sample, assessors recorded detectability (yes = 1/no = 0), free-text descriptors, and intensity (0–5). The group odor threshold in water was defined as the lowest concentration at which at least 50% of the panel reported detection (≥10 of 20 assessors answering “yes”). To quantify uncertainty in these group thresholds, we estimated 95% confidence intervals (CIs) by non-parametric bootstrap resampling of panelists. Specifically, for each compound we repeatedly resampled the 20 assessors with replacement (10,000 replicates), recomputed the group threshold for each bootstrap sample using the same ≥50% detection rule, and took the 2.5th and 97.5th percentiles of the resulting bootstrap distribution as the lower and upper bounds of the 95% CI. Thresholds are reported as the bootstrap median with 95% CI, expressed in mg/L.

All sensory thresholds were determined in a distilled-water matrix without sugars, acids, or juice pulp. Thresholds in real juice matrices may differ due to matrix effects (e.g., interactions with sugars, organic acids, or suspended solids); therefore, extrapolation of water-based thresholds to passion fruit juice should be made with caution.

## 3. Results

### 3.1. Data Collection

**Figure 1:**
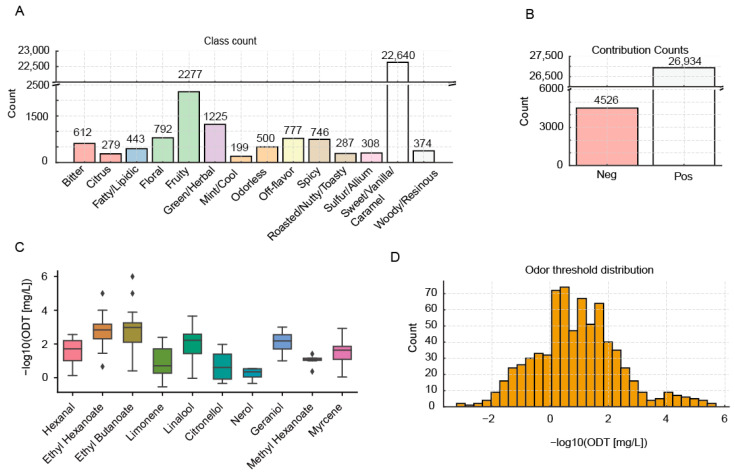
Overview of the odor contribution and odor threshold databases. (**A**): Bar chart of counts by odor class. (**B**): Bar chart of counts by odor class. (**C**): Boxplots of reported odor thresholds (−log10(ODT [mg/L])) for selected compounds. (**D**): Distribution of odor thresholds (−log10(ODT [mg/L])) across the odor threshold database.

We compiled flavor-descriptor annotations for 31,459 volatile compounds and grouped descriptors into higher-level sensory categories. Sweet/Vanilla/Caramel was the most abundant category (n = 22,640), followed by Fruity (n = 2277), Green/Herbal (n = 1225), and others (Figure 1A). Mapping categories to contribution labels yielded 26,934 positive-contribution compounds and 4526 negative-contribution compounds (Figure 1B). Because Sweet/Vanilla/Caramel and the positive class were far more prevalent than other categories/contributions, we downsampled during model training and used sample-weighted losses to mitigate label imbalance.

Odor threshold values for 716 volatiles were aggregated and standardized to mg/L. A random set of ten compounds spanning major chemical classes (esters, terpenes, aldehydes) illustrated that thresholds varied by several orders of magnitude (Figure 1C). To improve robustness and reduce the influence of outliers while retaining sensitivity to low-threshold odorants, we used the median of reported thresholds for each compound during model training. Thresholds were then transformed to a negative logarithmic scale (−log10), and the resulting distribution was approximately normal (Figure 1D).

### 3.2. Development and Validation of Volatile Aroma Contribution and Odor Thresholds Predictive Model

**Figure 2:**
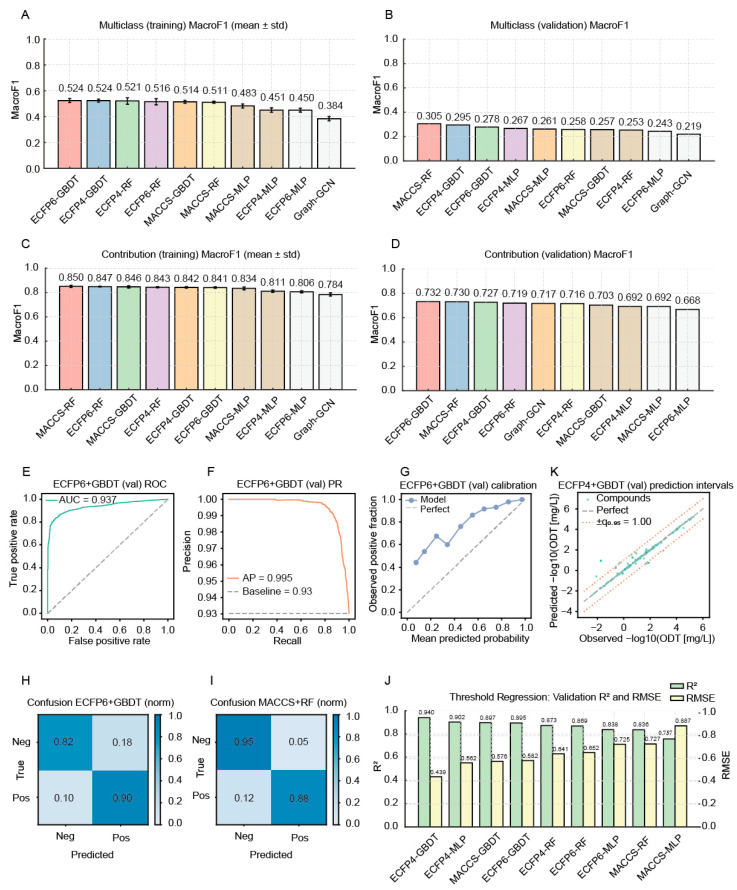
Evaluation of molecular fingerprint–model combinations for predicting odor category, odor contribution, and odor threshold. (**A**): Mean (±SD) macro-F1 from 5-fold cross-validation on the training set for odor category prediction; (**B**): Macro-F1 on the validation set for odor category prediction; (**C**): Mean (±SD) macro-F1 from 5-fold cross-validation on the training set for odor-contribution prediction; (**D**): Macro-F1 on the validation set for odor-contribution prediction; (**E**): Receiver operating characteristic (ROC) curve on the validation set for odor contribution prediction by the ECFP6–GBDT model; (**F**): Precision–recall (PR) curve on the validation set for odor contribution prediction by the ECFP6–GBDT model; (**G**): Calibration curve (observed vs. predicted positive fraction) on the validation set for odor contribution prediction by the ECFP6–GBDT model; (**H**): Normalized confusion matrix on the validation set for odor contribution prediction by the ECFP6–GBDT model; (**I**): Normalized confusion matrix on the validation set for odor contribution prediction by the MACCS–RF model; (**J**): Coefficient of determination (R^2^) and root-mean-square error (RMSE) on the validation set for odor threshold regression across fingerprint–model combinations; (**K**): Predicted versus observed −log10(odor threshold, ODT [mg/L]) for the ECFP4–GBDT model on the validation set, with the 1:1 line and ±1-log10-unit error bounds. Abbreviations: AP, average precision; AUC, area under the ROC curve; ECFP4, Extended-Connectivity Fingerprints (diameter 4); ECFP6, Extended-Connectivity Fingerprints (diameter 6); GBDT, Gradient Boosting Decision Trees; Macro-F1, macro-averaged F1 score; MACCS, Molecular ACCess System keys; ODT, odor detection threshold; PR, precision–recall; RF, Random Forest; RMSE, root-mean-square error; ROC, receiver operating characteristic; R^2^, coefficient of determination.

We first explored a multi-class flavor-category classifier. Across fingerprint–model pairs, we performed 5-fold cross-validation on the training set and selected hyperparameters by macro-F1. The best cross-validation result was achieved by the ECFP6–GBDT pair; however, macro-F1 remained modest (0.52; Figure 2A), and differences versus other GBDT and RF combinations were small (Supplementary Table S2). Because a Murcko scaffold split separated training and validation sets, validation performance more faithfully reflected generalization. After training with the selected hyperparameters, validation set results were as follows (Figure 2B): MACCS–RF achieved the highest macro-F1 (0.30) yet maintained high weighted-F1 (0.88) and accuracy (0.88) (Supplementary Table S3). A per-class analysis using MACCS–RF showed the best performance on Sweet/Vanilla/Caramel (precision 0.99, recall 0.98, F1 0.98), with substantially poorer performance on other classes, indicating that residual imbalance affected the classifier despite downsampling and class weighting. Given that a binary aroma contribution endpoint was sufficient for screening crop flavor compounds, we next trained a contribution classifier.

For contribution prediction, 5-fold cross-validation showed that MACCS–RF performed best (macro-F1 = 0.85; Figure 2C; Supplementary Table S4). On the held-out validation set, both ECFP6–GBDT and MACCS–RF performed well (macro-F1 = 0.73; Figure 2D; Supplementary Table S5). Receiver operating characteristic and precision–recall curves for the final ECFP6–GBDT model indicated strong discrimination despite the heavy class imbalance (AUC = 0.937; AP = 0.995, compared with a positive-class prevalence baseline of 0.93; Figure 2E,F). The calibration curve showed that predicted probabilities were slightly conservative at low scores but closely matched the observed fraction of positive contributors at higher scores (Figure 2G); the overall Brier score was 0.073, consistent with good probabilistic accuracy. Confusion matrices indicated predominantly correct calls for both models (Figure 2H,I). Per-contribution analyses showed high accuracy and recall for the positive-contribution class for both models, whereas recall for the negative-contribution class was high but accuracy was lower (Supplementary Tables S6 and S7), again reflecting the class imbalance. We therefore selected ECFP6–GBDT as the final contribution predictor because its validation macro-F1 was slightly higher than that of MACCS–RF; on the held-out validation set, ECFP6–GBDT achieved macro-F1 = 0.732, weighted-F1 = 0.912, accuracy = 0.896, macro-precision = 0.684, and macro-recall = 0.860.

For odor threshold regression, 5-fold cross-validation identified ECFP6–GBDT as the top performer (Supplementary Table S8). On the validation set, ECFP4–GBDT performed best (R^2^ = 0.94; RMSE = 0.44; Figure 2J) (Supplementary Table S9). Stratified evaluation by threshold tertiles showed the lowest error for low-threshold compounds (RMSE = 0.17), with larger errors for the mid and high tertiles (RMSE = 0.38 and 0.64). To further characterize predictive uncertainty, we constructed approximate 95% prediction intervals on the validation set using a simple residual-based conformal approach. The 95th percentile of absolute residuals was ~1.0 in −log10(ODT [mg/L]) units, and 94.4% of validation compounds fell within the ±q_0_._95_ band around the identity line (Figure 2K), close to the nominal 95% coverage, indicating that the intervals provide a reasonable summary of single-compound prediction uncertainty. Accordingly, we adopted ECFP4–GBDT as the final odor threshold predictor.

**Figure 3:**
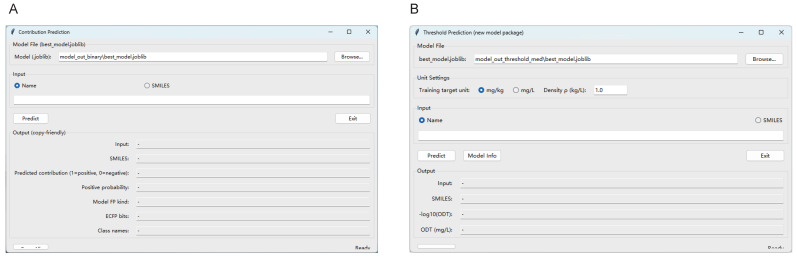
Graphical user interface (GUI) of odor contribution and odor threshold prediction models. (**A**): Odor contribution model. (**B**): Odor threshold model.

Both the aroma contribution and odor threshold models provided a graphical user interface (GUI; Figure 3) and a command-line interface (CLI). Users could supply either compound names or SMILES strings as inputs. By replacing the training dataset, the models could be retrained and re-evaluated, streamlining subsequent upgrades.

### 3.3. Predictive Models Revealed Novel Aroma Compound in Passion Fruit

We then applied the models to passion fruit juice profiled with the WTV 2 method, detecting 254 volatile compounds spanning alcohols, aldehydes, ketones, esters, and others (Supplementary Table S10). To benchmark against known odorants, we compared model outputs with database annotations. The flavor-contribution classifier predicted positive contribution for butanoic acid, 2-methylbutyl ester, *cis*-geraniol, and citronellol, consistent with PubChem and reported descriptors [29,33]. For odor thresholds, the regression model predicted a value of 0.0052 mg/L for 2-pentylfuran versus 0.006 mg/L reported (www.chemicalbook.com, accessed on 13 November 2025). For furfural, the reported value was 3.5 mg/L (https://www2.mst.dk/udgiv/publications/1999/87-7909-563-1/html/furfural/kap01.htm, accessed on 13 November 2025) and the model predicted 2.78 mg/L. Collectively, these comparisons indicated good predictive ability.

We then prioritized compounds detected in passion fruit that were absent from the training corpus and, to our knowledge, lacked published threshold/contribution entries: acetic acid, 2-phenylethyl ester and menthyl acetate. Using the classifier, 2-phenylethyl acetate was predicted to have a positive contribution (probability 94%), whereas menthyl acetate was predicted to be negative (probability 7%). The threshold model predicted odor thresholds of 0.37 and 0.39 mg/L for these two compounds, respectively. To validate these predictions, we determined odor thresholds and odor descriptors by serial dilution and sensory evaluation in water (n = 20; Table 1, Supplementary Table S11). For 2-phenylethyl acetate, the experimentally determined group threshold was 1.0 mg/L (bootstrap median, 95% CI: 0.10–1.0 mg/L), and 18 of 20 panelists detected the odor at this concentration. This CI encompasses the model-predicted threshold of 0.37 mg/L, and most panelists described the odor as floral, in agreement with the predicted positive contribution. For menthyl acetate, the group threshold was also estimated at 1.0 mg/L (95% CI: 1.0–10.0 mg/L), with exactly 10 of 20 panelists reporting detection at 1 mg/L. Although the model slightly underestimated the experimental threshold (0.39 vs. 1.0 mg/L), the prediction was within one order of magnitude, and panelists predominantly described the odor as grassy, consistent with the predicted negative contribution.

## 4. Discussion

Our aroma contribution classifier achieved accuracy on par with—or exceeding—descriptor-prediction approaches reported previously [21,23,24]. For the practical identification of aroma-active compounds, a binary “contributes vs. not” framing was often sufficient to prioritize impactful volatiles. Recasting multi-class descriptor prediction as a binary contribution task improved both accuracy and robustness when screening crop aroma constituents. Likewise, the odor threshold predictor performed comparably to related method [21]. Taken together, these results indicated that our models delivered high accuracy suitable for the efficient discovery and prioritization of crop aroma compounds. In addition, we provide a reproducible code framework for model training, validation, and application, enabling researchers to leverage higher-quality curated data and thereby further enhance model performance.

Future work will proceed along three directions. (1) Although our odor threshold predictor performed strongly, its generalization is likely constrained by the modest size of available datasets. To improve generalization, we will assemble substantially larger odor threshold corpora by standardizing threshold-evaluation protocols across laboratories, generating additional experimentally measured thresholds, and incorporating matrix-specific measurements. (2) While collapsing multi-class odor descriptors into a binary contribution label has clear utility for discovery, it constrains the model’s ability to apportion contributions to specific sensory qualities (e.g., fruity, grassy). The robustness of the flavor-descriptor dictionary and rule-based mapping also requires further validation across additional datasets and sensory panels. We will expand descriptor datasets via targeted sensory panels to mitigate class imbalance and label sparsity observed previously, evaluate multi-label formulations alongside the binary framing, and refine the descriptor dictionary and mapping rules. In parallel, we will explore transfer learning strategies that are fine-tuned on related models to strengthen both descriptor and threshold predictions [25]. (3) We did not yet perform detailed interpretability analyses (e.g., SHAP values, permutation importance, partial dependence/ICE plots) for the contribution and threshold models. We will conduct SHAP-based explainability analyses to identify which molecular features most influence predictions of aroma contribution and odor threshold, and we will perform Aroma Recombination and Omission Tests to validate the impact of newly identified compounds on passion fruit aroma. These aspects, together with more comprehensive interpretable modeling, matrix-specific threshold measurements, and refinement of the descriptor dictionary, will be the focus of future work.

## 5. Conclusions

In summary, we aggregated volatile aroma contribution and odor threshold information and trained machine-learning models that leveraged molecular fingerprints and physicochemical descriptors to predict both endpoints. Compared with traditional experimental determinations, our approach was low-cost and rapid, and our validation and application studies demonstrated effective identification of crop aroma compounds. These models provided a practical tool for dissecting crop aroma chemistry and supported genetic improvement of aroma traits in breeding programs.

## Figures and Tables

**Table 1:** Serial dilution and sensory evaluation results of acetic acid, 2-phenylethyl ester, and menthyl acetate.

Concentration (mg/L)	Detected (Yes/Total n)	Descriptors (Term: Count)	Intensity (Mean ± SD)
acetic acid, 2-phenylethyl ester			
0	0/20	None: 0	0.00 ± 0.00
0.1	6/20	sweet: 3; floral: 2; alcoholic: 1; fruity: 1; rose: 1; woody: 1	0.47 ± 0.77
1	18/20	floral: 10; cooling: 3; sweet: 3; minty: 2; rose: 2; alcoholic: 1; fermented: 1; fruity: 1; honey: 1; leafy: 1; sour: 1; woody: 1	1.82 ± 0.88
10	20/20	floral: 14; sweet: 5; alcoholic: 1; cooling: 1; fruity: 1; honey: 1; minty: 1; rose: 1; woody: 1	2.49 ± 0.81
100	20/20	floral: 15; sweet: 7; fruity: 4; rose: 4; alcoholic: 1; honey: 1; minty: 1	3.77 ± 0.53
1000	20/20	floral: 15; sweet: 7; fruity: 4; rose: 3; minty: 2; fermented: 1; honey: 1; pungent: 1	4.55 ± 0.60
menthyl acetate			
0	0/20	None: 0	0.00 ± 0.00
0.01	2/20	grassy: 1; sweet: 1	0.10 ± 0.31
0.1	5/20	grassy: 3; cooling: 1; honey: 1; minty: 1; other: 1	0.30 ± 0.55
1	10/20	grassy: 5; leaf: 2; other: 2; cooling: 1; honey: 1; leafy: 1; minty: 1	0.72 ± 0.87
10	18/20	grassy: 9; leaf: 8; fruity: 5; cooling: 2; honey: 1	2.06 ± 0.96
100	20/20	grassy: 17; leaf: 13; cooling: 9; other: 4; floral: 1; honey: 1; sour: 1	3.25 ± 0.87

## Data Availability

All code, curated datasets (SMILES/targets/splits), trained models, and full results are permanently archived on Zenodo at https://zenodo.org/records/17559514, accessed on 13 November 2025 (DOI available on record). A GitHub mirror is provided at https://github.com/yuanhonglun/odor_prediction_models, accessed on 13 November 2025 for issue tracking and incremental updates. The analyses are reproducible using the supplied environment files and scripts.

## Supplementary Materials

The following supporting information can be downloaded at https://www.mdpi.com/article/10.3390/metabo15110747/s1, Supplementary Table S1 Rules for category consolidation and contribution labeling; Supplementary Table S2 Five-fold cross-validation results (training set) for odor-class prediction across molecular fingerprint–model combinations at optimized hyperparameters; Supplementary Table S3 Validation set results for odor-class prediction across molecular fingerprint–model combinations at optimized hyperparameters; Supplementary Table S4 Five-fold cross-validation results (training set) for odor contribution prediction across molecular fingerprint–model combinations at optimized hyperparameters; Supplementary Table S5 Validation-set results for odor contribution prediction across molecular fingerprint–model combinations at optimized hyperparameters; Supplementary Table S6 Performance of the ECFP6–GBDT model in predicting different odor contributions; Supplementary Table S7 Performance of the RF-MACCS model in predicting different odor contributions; Supplementary Table S8 Five-fold cross-validation results (training set) for odor threshold prediction across molecular fingerprint–model combinations at optimized hyperparameters; Supplementary Table S9 Validation-set results for odor threshold prediction across molecular fingerprint–model combinations at optimized hyperparameters; Supplementary Table S10 Volatilome profile of passion fruit juice; Supplementary Table S11 Sensory evaluation results of acetic acid, 2-phenylethyl ester and menthyl acetate.
